# Two- and Three-Dimensional Culture Systems: Respiratory In Vitro Tissue Models for Chemical Screening and Risk-Based Decision Making

**DOI:** 10.3390/ph18010113

**Published:** 2025-01-16

**Authors:** Joanne Wallace, Mary C. McElroy, Mitchell Klausner, Richard Corley, Seyoum Ayehunie

**Affiliations:** 1 Charles River Laboratories, Edinburgh EH33 2NE, UK; mary.mcelroy@crl.com; 2 MatTek Corporation, Ashland, MA 01721, USA; mklausner@mattek.com (M.K.); sayehunie@mattek.com (S.A.); 3 Greek Creek Toxicokinetics Consulting LLC, Boise, ID 83714, USA; rcorley.gctc@gmail.com

**Keywords:** 2D and 3D lung models, inhaled chemical safety assessment, in vitro, human, rat, computational modeling, validation

## Abstract

Risk of lung damage from inhaled chemicals or substances has long been assessed using animal models. However, New Approach Methodologies (NAMs) that replace, reduce, and/or refine the use of animals in safety testing such as 2D and 3D cultures are increasingly being used to understand human-relevant toxicity responses and for the assessment of hazard identification. Here we review 2D and 3D lung models in terms of their application for inhalation toxicity assessment. We highlight a key case study for the Organization for Economic Cooperation and Development (OECD), in which a 3D model was used to assess human toxicity and replace the requirement for a 90-day inhalation toxicity study in rats. Finally, we consider the regulatory guidelines for the application of NAMs and potential use of different lung models for aerosol toxicity studies depending on the regulatory requirement/context of use.

## 1. Background

Risk to human health from the inhalation of aerosol chemicals can arise from occupational exposure, day-to-day use of products for plant and insect control, contaminated diet, drinking water, spray chemicals and disinfectants, and agrochemicals [1,2,3,4]. Of all the human organ systems, the respiratory tract is the most exposed organ to inhaled chemicals. For instance, the average adult human takes about 12–14 breaths/minute with an average tidal volume of 464–750 mL/breath and an intake of about 9354–12,960 L of air per day under resting conditions [5]. In this process, the respiratory tract is the point of first contact for chemical aerosols, vapors, and particulates [6].

The classical approach for investigating portal-of-entry effects from inhalation exposures has been in vivo laboratory animal inhalation studies. These studies require considerable numbers of animals and are costly and time-consuming. These factors, combined with significant anatomical and physiological differences between animal and human respiratory systems (including chemical deposition, obligate nose-only breathing in rodents, and species differences in breathing modes), make the translation of in vivo inhalation results into predicted human effects difficult [7,8].

However, New Approach Methodologies (NAMs) are increasingly being viewed as a complimentary/alternative approach to in vivo studies [9]. The term NAMs is a general phrase covering technologies, methodologies, approaches, or a combination thereof that can provide information on chemical hazard and risk assessment and avoid the use of animals. NAMs may include in silico, in chemico, in vitro, and ex vivo approaches used individually or in combination [10,11,12,13,14,15].

Here we review lung 2D and 3D in vitro cultures in terms of their application for human lung safety assessment and consider their relative advantages and limitations. We highlight an OECD case study using 3D organotypic lung cultures used to assess the hazard of a fungicide (chlorothalonil), which ultimately replaced a 90-day inhalation toxicology study in rats [16,17,18,19,20]. Finally, we review the current regulatory approach and consider how in vitro lung 2D and 3D models might work within this approach for the human-based inhalation safety assessment of aerosol exposure to chemicals.

## 2. Overview of Lung and Conducting Airway Structure and Function

The primary function of the lung is gas exchange, and this is achieved with structures within the conduction zone (bronchial region) and the respiratory zone (alveolar region) [21]. The human trachea and upper conducting airways are composed of a pseudostratified epithelium on the luminal side, with a basal lamina lined with mesenchyme, cartilage, smooth muscle, blood vessels, and immune cells (reviewed by Schiller et al., 2019 [22]). Aerosols that are deposited in the upper conducting airways (head through to bronchial region) may be cleared by several mechanisms depending upon the aerosol properties. For poorly soluble aerosols, mucociliary clearance is the dominant mechanism, although local immune cells such as dendritic cells can be involved to a lesser degree [5,23]. For soluble aerosols, dissolution and partitioning into local tissues or systemic blood circulation are additional mechanisms for conducting airway clearance.

The alveolar region is covered by very large, thin, alveolar epithelial type I cells, which are the main epithelial barrier to gas exchange. Alveolar epithelial type II cells form tight junctions with type I cells, are important for repair and surfactant regulation, and are involved in immune functions. Resident immune cells, particularly alveolar macrophages, also function to protect the lung by clearing aerosols that deposit in the alveolar region [23]. This process is considerably slower than the mucociliary clearance process in the upper conducting airways, which usually completes within 24–48 h, with poorly soluble particles being retained for days, weeks, or months depending upon their location and properties [23]. Macrophages are also important for coordinating the immune response, in combination with alveolar epithelial cells, to ensure an appropriate inflammatory process to inhaled particles. It has been estimated that there are over 40 different cell types in the human lung [22,24].

The conducting airways and extra thoracic region (particularly nasal and oral epithelia and larynx) can be a target site for inhaled chemical deposition and toxicity, as they form the entrance to the respiratory tract and as such are the first line of defense against airborne chemicals. As with the bronchioles, these tissues are lined by a stratified squamous epithelium of columnar cells and in some areas, a respiratory epithelium, with an underlying loose connective tissue containing serous–mucous glands [25].

## 3. Lung 2D Cultures

Monolayer or two-dimensional (2D) cell cultures have been used as an in vitro tool in toxicology, disease modeling, and pharmaceutical research for over a century. In 2D cell culture systems, monolayer cells or cell lines are cultured on plastic or glass surfaces and are the simplest forms of tissue culture. To date, much of the published literature describes studies using immortalized/transformed cell lines such as PTBE, BEAS-2B, A549, PSAE, Met-5A, 16HBE14o, and Calu-3 due to their accessibility, speed and ease of use, cost-effectiveness, and ability to combine with precise gene, protein, cytokine, metabolite, and enzyme analysis tools [26,27,28,29,30,31,32]. Refer to Table 1 for a summary of the most commonly used respiratory cell types and their advantages and limitations.

The differences between commonly used cell lines were examined in a comparison of upper airway (PTBE and BEAS-2B) and lower airway (A549, PSAE, MeT-5A and Calu-3) cell cultures derived from a range of transformed, cancer-derived, or primary cell sources [27]. The authors have identified differences in confluency, proliferation/metabolic rate, gene expression, and antioxidant capacity despite identical handling. These studies indicate that it is critical to select cell lines or sources where the signaling pathways relating to the target adverse effect are unaltered, as well as being derived from the relevant site of injury cells, to generate better translation to in vivo outcomes.

Primary human bronchial epithelial (HBEC) and alveolar epithelial cells from either healthy or diseased donor tissues can be viewed as a preferable option to continuous cell lines for safety assessment testing due to their non-cancerous/non-transformed origin and resemblance in morphology, organization, stratification, and physiological function to the human airway epithelium [50,51,52]. However, the ability to scale up is limited compared to immortalized cell lines. The isolation and purification of alveolar epithelial cells are very challenging—particularly alveolar type I cells, given their size and complex structure [53,54]. In this regard, iPSC-derived alveolar epithelial cells have emerged as an alternative source of cells to understand injury and repair in human lung safety assessments, although to date there has been limited use of iPSC-derived cells for safety assessments. Nonetheless, iPSC-derived alveolar epithelial type II cells have been developed to support SARS-CoV-2 infection, and these cells support many of the in vivo functions of alveolar type II cells such as viral entry, cellular responses to the pathogen, and viral replication. Therefore, iPSC holds much promise for an expandable and useful source of cells for other in vitro applications [54,55]. Two-dimensional (2D) tissue models have limitations in chemical screening: they lack complex tissue structures and do not fully recapitulate the physiology and barrier function of the native tissue. As a result, monolayer cell culture systems could be sensitive to chemical exposure and may not accurately predict physiological responses to chemical or drug treatments.

## 4. 3D Lung Models

Organotypic multi-cell-type three-dimensional (3D) models for specific regions of the airway have been developed and shown to be physiologically and functionally relevant models in predicting human respiratory tissue responses to chemical exposure. 3D models of the human lung have been widely used to assess inhalation hazard and risk. Currently available in vitro organotypic 3D human airway epithelial models are discussed below, along with a summary of their main advantages and limitations included in Table 2.

**Figure 1:**
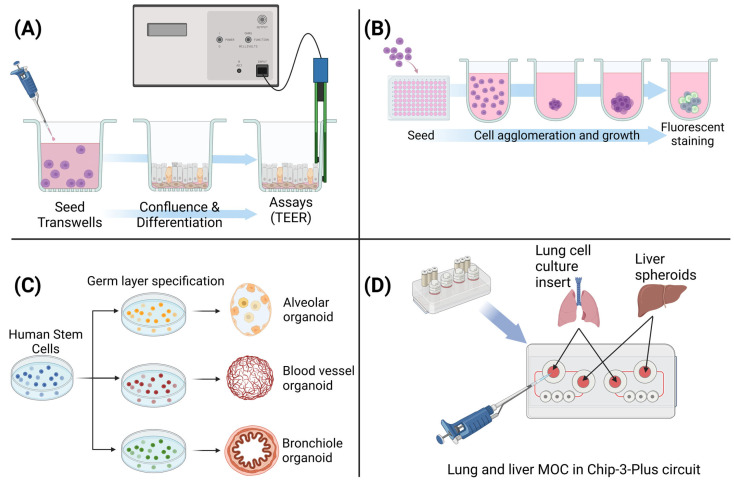
Summary of 3D lung models: (**A**) air–liquid interface organotypic tissue models, (**B**) spheroids, (**C**) organoids, and (**D**) multi-organ on a chip combining lung ALI tissues and liver spheroids on a multi-organ microfluidic device. Created in https://BioRender.com.

(1) Cell-line-based (Calu-3 cells): Calu-3 cell line co-cultured with fibroblasts under ALI conditions for 14 days have been shown to form a multilayered epithelium with adherence (E-cadherin) and tight junction (ZO-1) formation, marginal mucus production, and epithelial barrier formation [56]. However, the ability of the model for cilia formation and mucociliary clearance is controversial. The discrepancies could be associated with variations in growth conditions of the cells in different laboratories and differences in cell passage numbers used to make the tissue models. However, defective integrin signaling has been noted in Calu-3 cell-based tissue models, which may interfere with proper polarization to form 3D tissues [57].
(2) Spheroid cultures: Two methods have been used to generate spheroid cultures: (1) hanging drop and ultra-low attachment (ULA) methods. In the hanging drop method, cell suspensions of ~10 µL are placed on a flat surface of the culture plate wells, and then the surface of the droplets are turned upside down. This allows gravitational force to drag the cells to the bottom of the droplet to form aggregates or spheroids, the simplest of the 3D models. Such models have been used to study air toxicants such as benzene. While the system was able to recapitulate cell–cell interaction, the results obtained for air toxic exposure were sensitive and more variable compared to 2D cell culture systems [58]. However, the method is time-consuming and very tedious, which limits its wider application in high-throughput screening of chemicals. Since droplets can be far from the bottom of plates, imaging visualization becomes sub-optimal and challenging. To overcome issues associated with the hanging drop method, ultra-low attachment (ULA) plates were developed. These plates are created by coating the bottom of the wells of a cell culture plate with a non-adhesive material, which prevents cell adhesion and protein absorption. To these ULA plates, a cell suspension is added. Cells settle at the bottom of the well (but do not attach to the plate surface) and form spheroids. ULA plates are now commercially available from companies such as Corning Life Sciences (Tewksbury, MA, USA) and faCellitate (Mannheim, Germany). However, in general, spheroids do not mimic the microarchitecture and pathophysiology of the human lung, and are not good in vitro lung models for studying exposure to environmental factors, airborne pathogens, and therapeutic agents [59].
(3) Organoids: Organoids are derived from a population of stem cells (adult or pluripotent) [60] and are capable of maintaining stem cells during in vitro culture. During formation, organoids develop into 3D tissues that recapitulate in vivo-like bronchi/bronchioles and alveolar tissue structures [61,62], alveoli [63], and even multi-lineage structures [64]. Organoids play a valuable role in advancing our understanding of the respiratory system, but their use for inhalation studies has been limited due to the organoid lumen, which represents the apical surface of the lungs, develops inward, and is not accessible for direct chemical exposure. There have been attempts to mitigate this problem by applying the microinjection technique to deliver chemicals or therapeutics, mostly in intestinal organoids. However, in lung organoids, this technique has only been applied to propagate microorganisms or parasites [65]. Moreover, pluripotent stem-cell-based organoid cultures are immature and have difficulty in obtaining fully differentiated lung cell types [66].
(4) Multi-organ on a chip (MOC): MOCs are 3D tissue models in a microphysiological system in a flow format that makes organ–organ interaction or crosstalk possible. MOC provides the potential for preclinical studies to address the absorption, distribution, metabolism, excretion, and toxicity of substances in an in vitro microenvironment by co-culturing human three-dimensional organotypic cultures such the lung and liver spheroids on a chip. In one study, a 3D ALI bronchial MucilAir culture was co-cultured with liver spheroids in a Chip3plus platform (TissUse, Berlin Germany) to assess the potential toxicity of inhaled substances [67]. These models mimic human respiratory tract responses to inhaled chemicals in that they consider the influence of other organs on the chip in determining the toxicity levels of inhaled chemicals. An example of the TissUse MOC where two or more organs can be cultured under flow is shown in Figure 1. While this technology is a breakthrough in the field of advanced 3D tissue modeling, it is relatively expensive and is not in a high-throughput format. Currently there are over 40 different organs on chip platforms. The major players in microfluidic chip device production and applications include TissUse GmbH, Emulate, Inc., CN Bio Innovations, AlveoliX AG, MIMETAS, Nortis, SynVivo, The Charles Stark Draper Laboratory, Inc., Aim Biotech, and AxoSim. However, reproducibility comparisons among the different chips’ formats using the same cell type and analytical outcome measurements are lacking or not documented.

(5) Organotypic tissue models: In the past two decades, tremendous efforts have been put towards the development of a variety of 3D culture systems, as well as the adoption of 3D cell culture systems in various disciplines from drug discovery to toxicity studies. The 3D culture systems provide excellent in vitro platforms and allow for the study of cellular responses in a setting that resembles in vivo environments [68]. These 3D models increase the translational capacity of inhalation studies compared to 2D cultures or animal studies. The application of organotypic tissue models for drug and chemical inhalation and other studies has been previously summarized [69,70]. The commercially available in vitro 3D respiratory models include EpiAirway™, EpiAlveolar™, EpiNasal™ (MatTek Corporation, Ashland, MA, USA), MucilAir™, and SmallAir™ (Epithelix Sarl, Geneva, Switzerland) [71]. These models and their advantages and main limitations are summarized in Table 2.

**Table 2:** Main Characteristics of Commonly used 3D Cultures.

Organotypic Model Type Commonly Used	Origin	Advantages	Limitations	Examples of Applications for Respiratory Toxicity	References
EpiAirway/ MucilAir/ SmallAir	Primary Human bronchial epithelial cells—contain ciliated cells, goblet cells, actively produce mucus. Can also include fibroblasts. Available from diseased donors (COPD, asthma, smokers and cystic fibrosis).	Use primary tracheobronchial epithelial cells Functioning cilia and mucus layer. Form tight junctions and express cytochrome P450, transporters and form a 3D organotypic cultures with multiple different cell types. Histological scoring as performed in whole tissue samples is possible. Pool of 14 healthy donors (nasal MucilAir) Standardized, supplied with Certificate of Analysis. Release criteria based on TEER and morphology. Long term culture (up to one year). Physiological relevant exposures possible to both apical and basolateral surfaces	Cost, which in turn reduces replicate numbers/statistical power. Lack of immune cells and vasculature and physiological stretch (compared with in vivo)	Used to assess toxicity to cigarette Used to assess toxicity to cigarette smoke and electronic cigarettes, nanoparticles and metal oxides, multiwalled carbon nanotubes, chemicals, agrochemicals and diesel exhaust and pollutants	[17,28,29,30,31,32,33,72,73,74,75,76,77,78,79,80,81,82,83,84]
EpiAlveolar	Primary human alveolar cells	Barrier function Physiological, apical and basolateral exposure possible. Other cell types such as macrophages, fibroblasts, and endothelial cells can be incorporated and crosstalk between cells is possible.	Cost, which in turn reduces replicate numbers/statistical power. Cells demonstrated to express alveolar epithelial type II cell marker (prosurfactant C) but limited information on specific epithelial type I cells.	Used to assess carbon Nanotubes. Used in a case study for point-of-contact toxicity in the lung (1,3,-dichloropropene)	[85,86]
hAELVI	Primary alveolar type II cells transformed with lentiviral vectors: healthy human tissue taken from tumour resection surgery. Sex and age not stated.	Alveolar type I, extending phenotype and barrier properties	Limited data on phenotype-specific markers. Cells express one marker of alveolar type I cells (caveolin) and a type II cell marker (surfactant protein C) and therefore may represent a ‘transitional alveolar epithelial cell’ type.	Limited but include assessment of nanoparticle toxicity	[87,88]
^AX^iAECs	Primary human AECs isolated from resected lung tissue, immortalized with InscreeneX’ CI-Screen technology. Donor details not well defined	Well characterised phenotype comprised of both alveolar type I and type II like cells based on cell-selective markers. Form tight barrier and can be maintained long term cultures in physiological conditions (stretch/breathing, and co-culture)	Cost, which in turn reduces replicate numbers/statistical power. Limited data on culture stability i.e., the ratio between alveolar type I-like and alveolar type II-like cells	Limited—new cell line	[52,89]

### 3D Lung Models for Safety/Hazard Assessment

**Figure 2:**
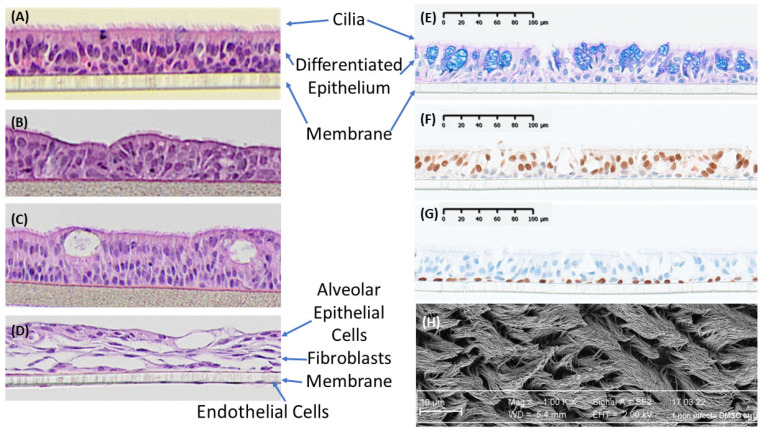
Structure of 3D airway and alveolar tissue models. Polarized and differentiated airway epithelia are shown for human EpiAirway (**A**), rat EpiAirway (**B**), rhesus monkey airway (**C**), human EpiAlveolar (**D**): (**A**–**D**) all at 200× magnification, MucilAir™ with Alcian blue H/E staining (**E**), MucilAir™ with FoxJ1 staining (ciliated cells: **F**), MucilAir™ with P63 staining (basal cells: **G**), and MucilAir™ healthy apical top view (SEM ×10K: **H**). MucilAir™ images are reproduced with permission from Epithelix Sarl.

Human upper airway air–liquid interface models are highly differentiated and well characterized in terms of their pseudostratified structure, cell types (mucus-producing goblet cells, ciliated cells with actively beating cilia, basal cells, and club cells), barrier properties, expression of tissue-relevant biomarkers, in vivo-like behavior, and cilia beating [29,90,91,92,93] and have been demonstrated to reflect the physiological conditions in the tracheal–bronchial region of the lung. Due to the emphasis on acute inhalation toxicity for risk assessment, there is increasing interest in the use of organotypic 3D airway models such as EpiAirway™ (Figure 2) and MucilAir™ for the determination of acute respiratory damage and contact irritancy. Such models are already being used to support in vivo testing by elucidating mechanistic processes, screening to deselect particularly toxic compounds or formulations, and predicting non-toxic starting doses and could eventually replace acute inhalation in vivo testing completely [11].

The 3D lung models are derived from healthy human donor airway cells seeded onto microporous membrane inserts and cultured under air–liquid interface (ALI) conditions for differentiation and stratification. The currently available 3D airway/lung models such as EpiAirway™ and MucilAir ^TM^ are produced from primary bronchial epithelial cells from healthy or diseased donors under Good Manufacturing Practice (GMP) conditions using standardized operation protocols (SOPs) and rigorous quality control standards to ensure long-term lot-to-lot reproducibility and reliable performance. EpiAirway™ has been successfully utilized by numerous research laboratories worldwide in applications including toxicology, drug delivery, pharmacology, and infectious disease research [72,73]. Similarly, MucilAir™ is derived from human airway cells collected from biopsies from diseased or healthy donors [92], the latter of which are also available as a pooled donor (pool of 14 donors) variant.

Lung organoids are also promising 3D models for chemical safety assessments. Lung organoids are self-organizing, three-dimensional culture systems derived from pluripotent stem cells. Consequently, they can develop, through co-culture with other cell types or via differentiation, into a structure that mimics the in vivo lung structure and function [94,95]. For example, human airway basal cells, when cultured in 3D Matrigel, form tracheospheres or bronchospheres and contain ciliated, goblet, and secretory cells. Human alveolar epithelial type II cells form alveolospheres with or without the support of fetal lung fibroblasts, whereas co-cultured alveolospheres may contain alveolar type I and II cells. There are limited data on the application of lung organoids for safety assessments; however, nanoparticle exposure to lung organoids was associated with the upregulation of inflammatory and tissue-remodeling genes [96]. Limitations of organoids include standardization and reproducibility; cells show an inward differentiation of the epithelial structure; also, the cells may not be fully differentiated compared to their in vivo counterparts.

## 5. In Vitro-to-In Vivo Exposures (IVIVE) Dosimetry Modeling

Determining appropriate dose metrics for IVIVE of toxicity observed from in vitro studies in respiratory cells creates a unique challenge for inhalation dosimetry modeling. For gases or vapors, human dosimetry models must integrate chemical properties such as concentration, exposure duration/profile, biochemical interactions with airway tissues (e.g., solubility, diffusivity, mucus–tissue–blood partitioning, metabolism, macromolecular interactions, perfusion clearance, etc.), along with human anatomy and physiology that drive airflows and material transport during inhalation and exhalation. This includes nasal and/or oral breathing under resting or working conditions. For in vitro systems, the method of exposure to gases or vapors should ideally reflect in vivo exposure methods rather than liquid (neat or solution) application to facilitate IVIVE dose metrics.

Aerosol exposures present additional complications associated with aerosol properties for in vivo exposures (e.g., size, shape, density, concentration, solubility, etc.), physical clearance properties (e.g., mucociliary clearance, phagocytosis via macrophages, dendritic cells, etc.), in addition to volatility and vapor pressure, which may be important to target tissue dosimetry including ADME (absorption, distribution, metabolism, elimination) processes. In vitro ALI aerosol exposures should ideally be carried out using atmospheric exposure systems rather than direct application. Before exposure systems such as Vitrocell^©^ (Vitrocell Systems GmbH, Waldkirch, Germany) became available, the direct application of aerosol material in a liquid vehicle suspension was a common practice, but it presents its own set of issues for IVIVE. For 2D lung models, the applied dose may not reflect the cellular dose since aerosol particles interact with surface media, bind to media macromolecules, and diffuse or sediment, depending upon aerosol properties, through the media to the cell surfaces. In these cases, in vitro dosimetry models such as ISDD (In Vitro Sedimentation, Diffusion, and Dosimetry) or ISD3 (In Vitro Sedimentation, Diffusion, and Dosimetry) may be necessary to determine in vitro cell doses that are suitable for IVIVE [97,98,99].

Irrespective of whether 2D or 3D lung models are used, the extrapolation of the in vitro applied dose to in vivo exposure is a key component in safety assessments. The extrapolation of results from in vitro studies to potential in vivo exposures (IVIVE) methods has been utilized for many years in human health risk assessments as the development of toxicokinetic and in vitro testing approaches have evolved [11,100,101,102]. As regulatory agencies have begun to call for well-validated alternative methods to traditional animal testing based upon in vitro studies with primary human cells, in silico approaches to IVIVE have become more broadly recognized as critical components. Two in silico approaches are leading platforms for IVIVE and cross-species comparisons for aerosol dosimetry that supplement prior physiologically based, pharmacokinetic (PBPK)-based approaches for other materials, target tissues, and routes of exposure. These two in silico approaches are computational fluid particle dynamics (CFPD) and the multiple path particle dosimetry (MPPD) models, which can be run independently or used in combination with each other or with other approaches (e.g., PBPK or NCRP (National Council on Radiation Protection Measurements) and ICRP (International Commission on Radiological Protection)) depending upon aerosol type, species, target tissues, or modes of action.

Both the CFPD and MPPD approaches are considered state-of-the-art for aerosol dosimetry and have been used by the EPA. Furthermore, each type of aerosol dosimetry model has its own strengths and weaknesses that complement each other when used together. The major advantage that CFPD models have over lower-dimensional aerosol dosimetry models is their realistic, 3D imaging-based airway geometries and well-established computational approaches that define airflows and aerosol fate and transport. Thus, CFPD models are ideally suited for predicting site-specific deposition of aerosols at high resolution within specific sites or airway regions [103,104]. However, such models do not generally extend into the deep lung due to the prohibitively computation-intensive nature of the simulations. As a result, current applications of CFPD models generally focus on the upper conducting airways (nose/mouth through tracheobronchial airways) and rely on other approaches to address pulmonary deposition.

MPPD, by comparison, is a detailed, one-dimensional mechanistic model that incorporates many of the complexities of species-specific anatomy, physiology, and aerosol physics that can predict aerosol deposition in individual airways of the tracheobronchial pulmonary regions. However, MPPD utilizes a simple empirical compartment to represent the entire head region (nose/mouth through larynx), with no ability to describe the site-specific deposition of aerosols in regions that have typically been identified as sensitive to contact irritation/cytotoxicity in short-term inhalation toxicity studies in rats. Thus, to provide the most accurate predictions, both approaches should be used in parallel to provide comparative site- (CFPD), airway- (CFPD and MPPD), and region-specific (MPPD) predictions of aerosol deposition in the full respiratory system.

MPPD has become one of the most widely used platforms for estimating aerosol dosimetry in multiple species and exposure profiles. While CFPD models require high-performance computing and specialized software and expertise, MPPD models have been publicly available for many years for use on desktop personal computers (ARA, https://www.ara.com/mppd/, accessed on 13 January 2025). As a result, the EPA is now completing its process of adopting an updated version of MPPD along with a Technical Support Document and User’s Guide to replace their empirical Regional Deposited Dose Ratio (RDDR) model following an external peer review in 2021. MPPD will facilitate the agency’s risk assessment process for aerosol exposures with more accurate predictions of regional and lung airway-specific aerosol deposition for a variety of aerosol properties and user-defined exposure conditions [19].

The key to effective IVIVE for risk assessments is the requirement of having a common “dose metric” that is relevant to the mode of action of the aerosol in both in vitro systems as well as in vivo models for extrapolating the target tissue dose to humans. For aerosols, this also involves controlling for key aerosol properties described above for in vitro studies if different from potential in vivo exposures (e.g., direct application vs. aerosol generating and exposure system such as Vitrocell^©^) vs. relevant human exposures and breathing scenarios. For example, the NAM approach used for chlorothalonil involved the use of a time-weighted average retained dose of aerosol in direct contact with specific sites in the human respiratory tract in human CFPD simulations vs. a benchmark dose analysis of time-dependent applied doses in vitro to human respiratory cells grown at the ALI, assuming occupational exposures via nasal and oral breathing under light exercise conditions. This dose metric is consistent with chlorothalonil’s contact irritation/cytotoxicity mode of action [16,18] as described in the following section.

A potential limitation of the “off-the-shelf” in silico MPPD/ or CPFD stand-alone platforms for IVIVE is that they are only capable of determining local deposition and site-of contact toxicity where exposures are driven by local dosimetry. Many chemicals and pharmaceuticals, although delivered via the lungs, will have modes of action that require partitioning into respiratory tissues and beyond, involving macromolecular interactions, or metabolism, as important key events for adverse responses. For these situations, peak or time-dependent target tissue/cell concentrations of a parent chemical or active metabolite that are affected by dose/exposure rate and clearance processes are typical metrics for IVIVE. However, these materials will require the development of material-specific in silico dosimetry models such as PBPK models [101,105,106] that are either directly coupled with CFD models [103,107,108] or combined with the use of aerosol dosimetry and PBPK approaches [105,109]. While templates based upon prior in silico models are available, this greatly increases the time and complexity of IVIVE and requires additional collaborative skill sets to both develop and interpret the data beyond simply conducting the in vitro study. Regardless, this process is critical for the successful application of NAMs in lieu of additional and potentially unnecessary animal studies. Collaborations at the earliest stage possible in conducting in vitro and in silico NAM approaches are thus highly recommended to enable the effective translation of in vitro studies to human health risk assessments.

Given the current limitations of conducting short-term in vitro toxicity studies in 2D or 3D organotypic respiratory cell systems, it is no surprise that measurable biomarkers of respiratory cell toxicity associated with key initiating events for acute toxicity modes of action or cytotoxicity/cell proliferative responses for longer-term exposures have received attention as NAM approaches have been developed [101]. For longer-term toxicity issues, more extensive, high-throughput batteries of in vitro assays including various “omics” and ADME measurements that serve to define the adverse outcome pathways (AOPs) and early key events as points of departure (PODs) that drive longer term or chronic AOPs in multiple systems are being investigated. Recent reviews by Andersen et al. [10], Chang et al. [102], and the Interagency Coordinating Committee on the Validation of Alternative Methods [9], among others, provide overviews of current concepts and guidance as continued NAM development, including broader systemic applications and exposures, is refined. Regardless, IVIVEs based upon pharmacokinetic or ADME processes for in vivo human exposures will be similar to previous extrapolations based upon PBPK, MPPD, CFD/CFPD, or combined in silico approaches.

## 6. 3D Tissue Model and Human-Relevant Exposure—OECD (2022) 31 Chlorothalonil Case Study

One of the most significant advances in the application of in vitro tissue-based methods for inhaled safety assessment is the regulatory decision to use an in vitro 3D lung model in combination with estimates of respiratory tissue exposures to assess the hazard from an inhaled pesticide—chlorothalonil. Specifically, the EPA accepted a NAM combining CFPD-based IVIVE modeling, real-world particle exposure characterizations, and benchmark dose (BMD) modeling of a contact cytotoxicity point of departure (POD) observed in human respiratory epithelial cell cultures exposed at the air–liquid interface (ALI). This NAM was accepted in lieu of additional rodent 90-day repeat dose inhalation toxicity testing, normally required for product re-registration [20]. This process was recently published as a case study by the OECD [30,110].

The NAM was based on the Source-to-Outcome framework for context proposed by the Computational Toxicology Program 2003 workshop of the USEPA (Source, Exposure, Dosimetry, Outcome and Risk Assessments) [111]. Under this framework, the Source was approved chlorothalonil-containing products following approved used and label directions. Exposure incorporated data from personal air-monitoring samplers carried by workers and provided data on particle size distribution and operator breathing measurements [112]. The Dosimetry aspect of the framework was a CPFD strategy [16], with the human clearance model calculating human upper airway deposition for the above scenarios. The next level of the NAM’s framework comprised Outcome data derived from in vitro measurements from 24 h exposure of MucilAir™ to a direct application (apical, 90.9 µL/cm^2^) of suspensions of the formulated fungicide chlorothalonil (Bravo 720), followed by measurements of TEER (tight junction integrity), lactate dehydrogenase (LDH) release (plasma membrane integrity), and resazurin metabolism (metabolic activity) [17]. The resulting in vitro point of departure was used to calculate human equivalent concentrations (using benchmark dose (BMD) modeling with USEPA BMDS software). Risk Assessments (the final arm of the framework) were conducted by comparing the in vitro-derived HECs to the previously determined potential exposure levels [18]. The conclusion was that the results compellingly verify the applicability of this testing strategy as an IATA (Integrated Approach to Testing and Assessment) for the identification of safety respiratory toxicants in operator exposure risk assessments and was subsequently used to grant a waiver from performing rodent 90-day repeat dose testing [20,110].

The peer review of the NAM by the Scientific Advisory Panel [110] recommended several pertinent points for future strategies. They suggested the use of pooled donor tissues if available, or multiple single donors if not, and sufficiently large numbers of tissue replicates; and since the primary endpoint of the in vivo tests being replaced is pathology, to include that in the in vitro tests, as the biomarkers used can prove overly sensitive. Finally, selected endpoints should relate to the adverse outcome pathways (AOPs) or any known target site or portal-of-entry toxicities that have been observed elsewhere. It is worth highlighting that target site exposure and point-of-entry toxicity have been highlighted as key events in inhalation toxicity AOPs [11].

## 7. Beyond the OECD Case Study Towards Formal OECD Validation Status

### Test Panels to Predict Hazard Categories for Chemical Inhalation Hazard from 3D Respiratory Models

The development of well-defined chemical test panels provides evidence to support the validation and robustness of in vitro systems. The earliest such testing included a test panel comprising 19 chemicals with a range of toxicities tested concurrently in EpiAirway™ (3 h), MucilAir™ (24 h), and A549 monoculture (24 h) [33]. Exposure was via submergence (monoculture) or ALI (organotypic) direct application. The concentrations that inhibited viability by 50% (IC50) were compared to rat 4 h LC50 values classified according to Environmental Protection Agency (EPA) and Globally Harmonized System GHS hazard categories. Sensitivities of 87.5–100% and specificities of 56–89% were obtained; however, only a modest ability to distinguish between toxicity subcategories was reported. The authors noted the difficulty in extrapolating to in vivo data, which in some instances was based on a lethal dose (LD)-50 of greater than a certain concentration, arising when insufficient animals died at the highest concentration. In addition, in vivo test classifications differed depending on application of vapor, dust, or mist, which further hindered predictions. Furthermore, there are genetic differences between the human in vitro tissue models and the in vivo rats, which makes the translation of animal results to predict human responses to chemical exposure difficult.

Subsequently, Jackson et al., 2018 [72] tested a larger panel of 59 chemicals with a broad range of modes of action and toxicities using EpiAirway™ for 3 h of exposure (direct ALI liquid exposure), followed by the MTT assay (which assesses toxicity directly by the rate of conversion of MTT into MTT formazan by actively metabolizing cells). A prediction model was created based on IC25 values to classify to GHS or EPA categories, and this was broadly shown to be promising, with good sensitivity (100%) and specificity (43.1% and 50%) for GHS and EPA, respectively, for acute inhalation Category 1 and 2 predictions. Improving the specificity of GHS/EPA categorizations by 3D in vitro tissue models is a potential gap that may have to be addressed in future research.

A NAM to identify local acute irritation in the skin, eye, and respiratory tract was presented by Kluxen et al., 2022 [74]. The respiratory arm of testing utilized rat EpiAirway™, with single and repeat exposures (direct ALI application of liquids) for two pesticides known to cause local acute irritation and correctly predicted the inhalation hazard. In addition, the broader data set indicated potential species differences regarding acute toxicity responses for some target sites, for example the eyes, limiting the value of in vivo animal data.

Recently, the authors compared 14 chemicals using the rat and human EpiAirway™ models, assessing intra- and inter-laboratory as well as inter-species differences at Charles River and MatTek [73] using the endpoint of IC25 for the TEER and MTT assays. The aim was to facilitate translational comparisons between in vitro human, in vitro rat, and in vivo rat data. The study showed good concordance between the species models in both laboratories, with a correlation coefficient (R^2^) of 0.78 and 0.88, respectively, when analyzed by TEER; and an R^2^ of 0.92 for both when analyzed by MTT. These results indicate that rat and human airway epithelial tissues respond similarly to acute exposures to these chemicals, and that rat EpiAirway™ will help extrapolate to in vivo rat toxicity responses and support screening as part of a 3Rs or NAM program. However, more expanded study is needed to further strengthen the initial findings in the 3D rat and 3D human airway tissue models.

## 8. Comparison of 2D and 3D Lung Models for Safety Assessment/Role of 3D Human Airway Models to Study Dose Application Routes In Vitro

Both 2D and 3D models have been suggested to be of value for predicting human health outcomes [28,31] depending on the phase of a tiered testing strategy. The advantages and limitations of 2D and 3D lung models have been summarized earlier in Table 1 and Table 2. Because of their ease of use, 2D models may be used to perform preliminary toxicology screenings and dose selections for more expensive 3D models. Nonetheless, the extrapolation of doses and effects between the 2D and 3D models is complicated by the fact that exposure is very different between the models, i.e., submerged vs. directed at the apical or basal surface, and there are also differences in terms of the rate and/or kinetics of deposition. In general, 3D models are more robust and less susceptible to toxicity than their 2D counterparts [28,29,31,32] due to a number of factors. Lower (regenerative) basal cell layers are protected from apical exposures by the pseudostratified epithelium, functional cilia, and secreted mucus [113]. Due to their polarized membrane and architectural features that mimic their in vivo counterparts, repeated/chronic exposures in vitro are also possible to replicate in organotypic models [28,30,32,60,75,110]. Three example case studies below highlight the importance of tailoring exposure conditions for distinct chemicals using 2D and 3D cultures.

Methyl Iodide: Methyl iodide is a reactive volatile chemical intermediate in the synthesis of some pharmaceuticals and pesticides and causes acute lung irritation/damage following inhalation exposure. Methyl iodide causes glutathione depletion resulting in oxidative stress and damage to the cells at the point of contact [114]. Mistry et al. [28] compared the toxicity of methyl iodide in 3D airway cultures (EpiAirway™) and BEAS-2B bronchial epithelial cells; methyl iodide exposure was performed both in solution and the ALI vapor route (using a Vitrocell 12/12 exposure system for 3 h). As found by others, the 3D cultures were more resistant to toxicity compared with the 2D culture system. In a subsequent repeat-exposure vapor experiment with EpiAirway™ (3 h per day for 5 days), cumulative effects on toxicity were observed at the lower concentrations of methyl iodide tested. However, the only analyses performed in this series of experiments were TEER and LDH release, with no examination of gene expression, inflammatory, or morphological changes. It is difficult to directly compare the data from the in-solution and vapor experiments due to the methyl iodide concentration for each being determined as a molar concentration or ppm, respectively. The team also found that the volatility of methyl iodide made dosimetry challenging to accurately determine and control. Finally, liquid application at the air–liquid interface has been shown to affect the bronchial epithelial transcriptome and other biological pathways [115], which might be specific to the method of delivery rather than the test chemical. This is an important paper demonstrating that the method of exposure to lung cells in vitro can also impact fundamental biological processes in the lung epithelium. That said, lungs have evolved to minimize fluid in the airspaces and maintain the airspaces free for gas exchange. It will be important to understand, in both airways and the alveolar epithelium, what is the normal response to fluid application. Nonetheless, this research highlights the challenges involved in comparing different aerosol doses due to varying equipment, conditions, exposure scenarios, and methods of expressing the delivered dose [28,75,113]. Consistent dosimetry terminology and reporting standards are required to allow for a direct comparison.

Nanoparticles: Nanoparticles are present in many industrial, consumer, and medical products, resulting in a dramatic increase in human exposure in recent times. The toxicity of nanoparticles depends on their type and characteristics, but a major feature is their small size (1 to 100 nm^2^), such that they are likely to deposit in the alveolar region and potentially remain in the lung and/or transfer to circulation, resulting in systemic exposure [116]. Some commercially produced organotypic alveolar models are available (EpiAlveolar™, MatTek [85]), but they do not fully reflect the alveolar barrier morphology of large-surface-area flattened type 1 and cuboid type 2 pneumocytes or the characterization of cell-specific markers. Frequently, the A549 cell line (an adenocarcinoma cell line originally derived from type 2 pneumocytes) is used to assess nanoparticle toxicity either alone or in co-culture [75]. However, to apply nanoparticles to this cell line requires a submerged delivery of the dose. This is likely to change the physicochemical properties (and agglomeration/dispersion/cell media interactions) of many particle types and therefore will impact their delivery to cells and thus, their toxicity profiles [99]. However, progress has been made in the form of the modular air–liquid interface aerosol exposure system (MALIES)—this system delivers a dry nanoparticle aerosol exposure of “alveolar-like” wells lined by cells; the exposure well is configured as a cleft, which helps to maintain optimum humidity and therefore prevent drying of the epithelial cell layer [117]. This nanoparticle example highlights that experimental conditions have to be modeled to reflect human exposure and consequently may require specialized equipment that is ideally generally available and peer-reviewed to aid in confidence building prior to regulatory use in in vitro safety assessment studies.

Silanes: Triethoxysilane (TES) and trimethoxysilane (TMS) belong to the organic silane family and are used as precursors to complex functional silanes, which find applications in multiple industries as surface coatings, adhesion promoters, sealants, and others. TES and TMS become vapors during the manufacturing process, and toxicity is possible in the case of an accident or if personal protective equipment recommendations are not followed [31]. While both silanes are acutely toxic when inhaled, they hydrolyze rapidly in water and the toxicity of their hydrolysis products is low, with no known inhalation toxicity (see [31] for review). Therefore, to evaluate and compare the in vitro toxicity of TES and TMS in 2D (BEAS-2B cell line) and 3D cultures (MucilAir™), Sharma et al. [31] generated silane vapors and diluted them with non-humidified nitrogen gas for aerosol exposures (i.e., dry aerosol). BEAS-2B experiments ran for 24 h following exposure, while MucilAir™ tissues also underwent a post-exposure recovery period of up to 7 days depending on the treatment group. As with other cited publications, the 2D cell line (BEAS-2B cells) was more sensitive than the polarized and well-stratified 3D tissue model (MucilAir™) to silane toxicity. Furthermore, the 3D tissues were also found to be advantageous for the assessment of additional endpoints such as TEER, cilia beating, and morphology changes (H&E). For example, the 3D tissues were able to “recover” from the initial insult as measured by the recovery in barrier integrity at day 7, but the morphology of the MucilAir cultures was not considered normal by the authors (although no specific assessment was reported in the publication). Nonetheless, this publication demonstrates that specific inhalation methods are required for reactive point-of-contact toxicants, and the toxicity of the products should also be considered for in vitro exposure. The paper by Sharma et al. also illustrates the potential value of the 3D cultures to explore the value of different biomarkers for the assessment of airway epithelial recovery.

## 9. Guidelines for the Regulatory Acceptance of NAMs

Several key reviews have been recently published on how to accelerate the regulatory acceptance of NAMs [9,10,15]. A common theme through these reviews is that the extent of validation should be fit-for-purpose and linked to the context of use (CoU). The review by Andersen et al. [10] proposed a tiered approach with progression through a tiered testing strategy governed by the decision context. Depending on the required risk assessment and margin of exposure, a decision maker might regard the information at any specific level to be sufficient. The Andersen et al. [10] tiered approach moves from computational model screening (Tier 1) to high-throughput in vitro screening (Tier 2), followed by fit-for-purpose assays (Tier 3) and finally bespoke complex assays, including microfluidic systems and organs on chips (Tier 4). Each tier is linked to relevant models of human exposure, i.e., quantitative in vitro to in vivo extrapolation (QIVIVE) to derive human equivalent exposures [10].

**Figure 3:**
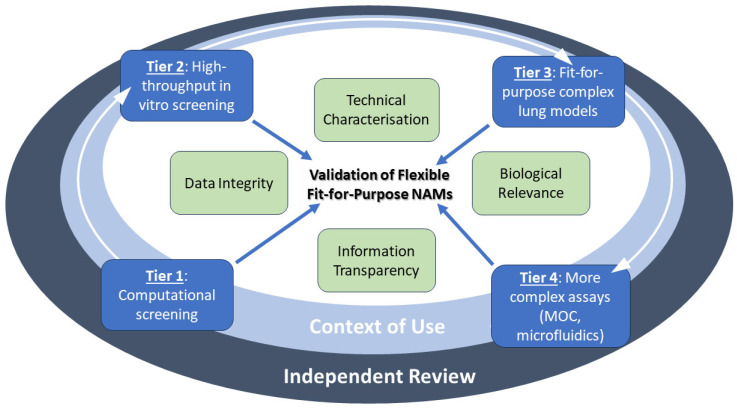
Composite framework for regulatory acceptance of NAMs, combining the tiered context-of-use strategy defined in Andersen et al. [10] and the six pillars identified by van der Zalm et al., 2022 [15]. The white arrows indicate the general direction of the experimental approach and increase in complexity with each tier. The blue arrows indicate that the level of validation is fit-for-purpose for the stage of assessment.

The ICCVAM NAM validation, qualification, and regulatory acceptance framework is more generalized [9], and the tiered approach fits well within the structure in terms of helping to define the context of use (Figure 3). A key idea within the ICCVAM framework is the idea of flexible, fit-for-purpose NAMs and not a one-size-fits-all approach. The framework is made up of six elements: defined context of use, biological relevance, data integrity, technical characterization, information transparency, and independent review [15]. Importantly, the guideline recognizes that human-relevant NAMs may provide mechanistic data for human risk assessments such that a comparison with animal data is unnecessary or even not helpful. The guideline highly recommends early interactions between NAM developers and regulatory agencies to ensure that the criteria for a particular CoU are understood and developed for the required regulatory need.

In the multi-level strategy for using new alternative methods and higher-throughput exposure tools for the context-dependent safety assessment cascade proposed by Andersen et al., 2019 [10], different in vitro lung models and methods of chemical application will be appropriate at different levels. Immortalized/transformed lung cell lines may have applicability in Anderson et al. Tier 2 for high-throughput assays of compounds with known mechanisms of action (for example) coupled with high-throughput in vitro–in vivo extrapolation. Submerged cell lines can be of value to perform initial dose range determinations prior to moving to ALI models [28], particularly for water-soluble (or at least sufficiently miscible) test compounds. However, initial and unpublished observations using a primary bronchial epithelial and fibroblast ALI co-culture model was shown to respond differently to a “liquid application”, where 120 µL medium (equivalent to 106 µL/cm^2^) was applied apically for up to 24 h and compared to cultures left at the ALI [115] (preprint) with regards to many biomarkers including gene expression pattern and barrier function (TEER and FD20 permeation).

On the other hand, it is generally accepted that in most scenarios, dose application via the aerosol phase (or dust, gas or vapor as applicable) is the most physiologically relevant application route as it closely reflects the in vivo scenario and is therefore more applicable at Tier 3 or 4 in the Anderson et al. scheme. For such applications, 3D organotypic tissue models play a significant role as shown by researchers of cigarette smoke and e-cigarette vapors [76,93,118,119] and pollutants [29,30]. However, aerosol application to 3D in vitro tissue models requires significant expertise and specialized equipment and is therefore lower-throughput and incurs considerably greater expense compared to direct liquid application.

The frameworks mentioned above are key for the conceptualization of risk assessment; however, developing relevant protocols and approaches for specific contexts of use will need to be developed for regulatory review. As an indication of what is required, the EPA has issued a Toxic Substances Control Act (TSCA) test for trifluoro (trifluoromethyl)oxirane) to better understand the health risks [120]. This test order states that the use of human primary cell culture models can be used for portal-of-entry studies examining single acute exposures as well as short-term repeat studies combined with the weight of scientific evidence approaches. Key elements of the TSCA Test Order are listed in Table 3 and include considerations that may require to be addressed for in vitro inhalation risk assessment in general terms.

## 10. Conclusions—Significance and Data Gaps

**Figure 4:**
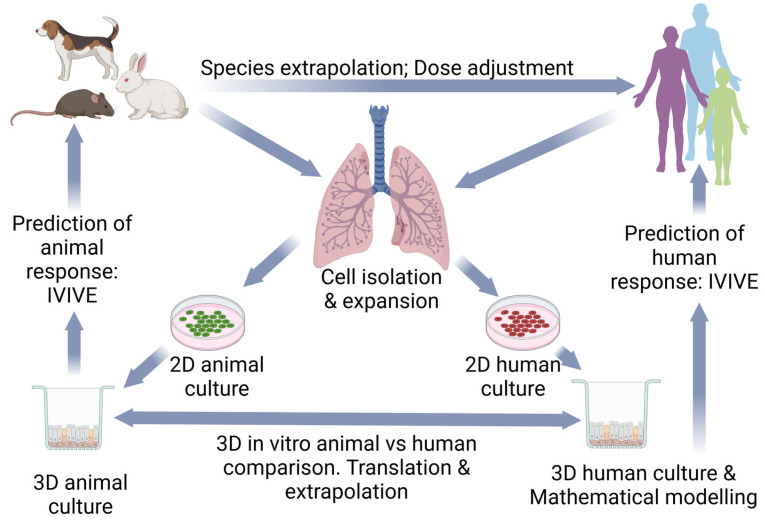
Evaluation of human inhaled risk is predominantly based on the use of animal models to assess no-adverse-effect levels. However, advances in human lung cell biology and computational modeling means that human-based risk assessments may improve if data from human in vitro models are part of the risk assessment process. In addition, well-curated animal model data can be used to validate in vitro models from animals to help establish confidence in NAM-based approaches in general. Created in https://BioRender.com.

Utilizing more physiological human in vitro lung models and in vitro tissue-based assay methods and prediction models may narrow the knowledge gap to screen inhaled aerosol chemicals for human hazard identification and risk assessment. While 2D models may have the potential for early screening, it is expected that 3D lung models, in combination with computational modeling, will have greater relevance for human lung risk assessment (Figure 4). However, much remains to be developed in terms of protocol designs for specific contexts of use including dose application and extrapolation to human exposure. That said, the EPA reviews ~500 new pesticide formulations each year and requires manufacturers to provide data from acute animal toxicity studies (commonly referred to as the “six-pack” of animal tests to assess dermal, oral, and inhalation toxicity, skin and eye irritation, and skin sensitization), which require >50 animals/formulation. Replacing the six-pack assays with fast and efficient in vitro cell-culture-based assays would reduce the number of animals used by up to 20,000 per year (https://cen.acs.org/environment/pesticides/US-EPA-struggles-replace-animal/97/i20, accessed on 13 January 2025). Hence, 3D in vitro human primary cell-based organotypic culture model assay systems can serve as alternatives to animal testing for aerosol chemical screening.

## Figures and Tables

**Table 1:** Main Characteristics of Commonly used 2D Cultures.

Cell Type Commonly Used	Origin	Advantages	Limitations	Examples of Applications for Respiratory Toxicity	References
A549	Human adenocarcinomic alveolar basal epithelial cell This cell line was developed by D. J. Giard et al. in 1972 by removing and culturing pulmonary carcinoma tissue from the explanted tumor of a 58-year-old caucasian male.	High throughput, easy to culture Secrete lung surfactant—important to reduce surface tension during breathing and prevent alveolar collapse. Can be grown in the absence of FBS.	Cell types/donors—single cell type therefore limited. Dysregulated cell cycle due to cancer origin. Do not form tight junctions and therefore barrier property cannot be assessed. Batch-to-batch variability of cells and media shown to affect key results	Used to assess toxicity to cigarette smoke, nanoparticles, chemicals, pollutants, and nanoparticles	[29,31,32,33,34,35]
BEAS-2B	Immortalised normal bronchial epithelial cells transformed with SV40/adenovirus 12 hybrid and cloned. Details of the donor not recorded in publication.	Differentiate into squamous cells and therefore have value for screening agents that affect cell differentiation. Share characteristic of human mesenchymal stem cells.	Cell types/donors—single cell type therefore limited. dysregulated cell cycle due to immortalisation. Do not form tight junctions Phenotype changes induced in BEAS-2B by FBS suggest that culture conditions should be carefully considered when using BEAS-2B as an experimental model Cultured cells can have two morphological cell types with 40% abnormal karyotypes.	Dust extracts Electronic cigarette Diesel Chemicals	[28,30,36,37,38,39,40]
Calu-3	Calu-3 cells were isolated from the pleural effusion of a 25-year-old Caucasian male with a lung adenocarcinoma https://www.cellosaurus.org/CVCL_0609. accessed on 13 January 2025	Produce mucus and form tight junctions (assessed by microscopy, transepithelial electrical resistance (TEER) and ZO-1 staining) and express cystic fibrosis transmembrane conductance regulator (CFTR)	Cell types/donors—single cell type therefore limited. Dysregulated cell cycle due to cancer origin. Grow in colonies rather than uniform monolayers. Culture conditions shown to affect morphology, barrier functions and drug transporter expression	Nanoparticles as pharmaceutical carriers	[26,27,41,42,43,44]
16HBE14o	16HBE14o- is a human bronchial epithelial cell line isolated from a 1-year old male heart-lung patient and immortalized with the origin-of-replication defective SV40 plasmid (pSVori-).	Express cytokeratin and form tight junctions and maintain directional ion transport. Cilia present when grown at an air-liquid interface. 16HBE14o- expresses high levels of cystic fibrosis transmembrane conductance regulator (CFTR) mRNA Spontaneous decline in barrier function due to increased transcellular conductance involving the CFTR channel	Cell types/donors—single cell type therefore limited. Do not form tight junctions Heterogenous depending on culture conditions	Used to assess nanoparticle toxicity nanotubules, gasoline exhausts	[45,46,47,48,49]
PTBE	Primary tracheal/bronchial epithelial cells	Will grow isolated from one another and become quiescent if 100% confluency is reached	Slow/limited growth compared to transformed or cancerous cell lines		[27]
PSAE	Primary small airway epithelial cells	Creates uniform monolayers with rounded cytoskeletons	Slow/limited growth compared to transformed or cancerous cell lines		[27]
Met-5A	Met-5A are lung (lower airway) mesothelial pRSV-T plasmid transfected cells	Create uniform monolayers	Elongated cytoskeleton, less metabolically active and proliferate faster than non-transformed counterparts. dysregulated gene/protein expression compared to primary cell types.		[27]

**Table 3:** Review of the Toxic Substance Control Act (TSCA)—Test Order and Considerations for the Future Amendments for Chemical Risk Assessment.

Element	EPA TSCA Proposal—Cell Culture Models Are Suitable for Examining Portal-of-Entry Effects from Single (Acute) Exposures as well as Short-Term Repeated Exposures—Key Requirements	Comment on Key Requirements (Generalised, All Chemicals)
Test System	Objective of Single Exposure Study (4 h) The purpose of this study is two-fold: to observe the effects of a single acute exposure on respiratory tract epithelial cells for integration with acute animal inhalation toxicity experiments in an IATA, and to select concentrations that do not cause excessive cytotoxicity for a subsequent repeated-exposure system	Considerations will be required around which airway model is most appropriate based on possible human exposure and what is known around test item aerodynamic particle size for lung deposition i.e., upper airways, lower airways or both.
Donor Tissue and Quality	General considerations: Study must involve primary respiratory epithelial cell cultures from at least 5 human donors, the cell cultures must have undergone mucociliary differentiation to ALI. The number of cell doublings from the donor must be reported and matched/consistent in studies. Donor tissue quality control and characterization: No exposure 3 inserts (wells) of tissues from each donor performed in triplicate Hematoxylin and eosin stain (H&E) to characterize and identify cells by shape, structure and organization within the epithelial culture Immunohistochemistry (IHC) for tumor protein 63 (p63), Mucin 5AC, Oligomeric Mucus/Gel-Forming (MUC5AC) for extracellular matrix composition and organization, and Forkhead Box J1 (FOXJ1) for ciliary expression Cell viability (tetrazolium salt, 2-[2-methoxy-4-nitrophenyl]-3-[4-nitrophenyl]-5- [2,4-disulfophenyl]-2H-tetrazolium (or WST-8), viability assay and lactate dehydrogenase (LDH) release Pro-inflammation (cytokine release)	If using a commercially produced model such as EpiAirway or MucilAir, production is certified and release criteria are reproducible to a very high level. It is sufficient that these parameters (including doublings and donor details) are certified at source, for each production batch, rather than per study. Current certification parameters can include TEER, cilia beat frequency, morphology and/or presence of a mucus layer. Note that multi-donor options are also available for some upper airway models, which are an acceptable alternative. For models produced in house, acceptance criteria need to be defined and may differ between models. Production standards must be high quality and reproducible. Other release criteria may be needed in the future as respiratory NAMs are more widely accepted e.g., cell-type specific markers to qualify the ratio between different cell types in the culture.
Single Acute Study	Study Design Details Six test article concentrations for the dose response assessment with N = 3 biological replicates for each of 5 donors. Different endpoints will also require biological replicates Test article exposures should be conducted while maintaining ALI conditions	Selection of test article concentrations will have to be justified and based on expected exposures in humans. Test article exposure and recovery time will need to be justified based on the context of exposure. Transient effects shortly after dosing for sensitive endpoints such as TEER could falsely indicate toxicity which may quickly recover and not lead to any downstream changes in the tissues. Also, the toxicity may take a certain amount of time for cellular proteins to take full effect. Allowing a recovery period after exposure would be beneficial to the risk assessment.
Dose Application	Not specified in the TSCA Test Order	The TSCA test order related specifically to assessment of a volatile gas (HFPO: vapor pressure is 5107 mmHg). There are many challenges associated with delivering this material to ALI cell culture. Specialist equipment and skills are required, with development and optimization in advance. Direct liquid application should be considered as an option, allowing greater control of deposited dose, in addition to shorter timelines and reduced cost. Maintenance of the ALI will be achieved by adding a small volume in a liquid format (90 µL/cm^2^). For volatile materials, consideration of whether inserts should be sealed/capped during exposure to prevent volatilization and cross-contamination of neighboring inserts. It has yet to determined what is the best dosing approach, which may depend on the test article. Different types of inhaled toxicants (e.g., gases, particles, fibers, aerosol delivered chemicals) might need different in vitro testing approaches and dosimetry methods, which remain to be determined.
Dose Metric	Not specified in the TSCA Test Order	Dose metric is a key parameter to report to allow comparison with other data. While liquid doses may be developed and analysed as a concentration (i.e., mg/mL etc), a mass per unit surface area can be more helpful to facilitate translation between models, and between in vitro and in vivo. Similarly, consideration should be given to determination of aerosol or gas concentrations, and the difficulties converting between ppm and mg/L air (for instance). Where possible, it is most helpful to discuss the deposited dose (mass per unit surface area).
Deposited Dose Determination	Not specified in the TSCA Test Order	Quantification of applied dose would be challenging, as would the subsequent PBPK modelling required to extrapolate human exposure levels if required. The dose route, control of volatility, and quantification of dosimetry should be considered in the test order to allow effective testing but are not specified.
End-point Assessment	Transepithelial/transendothelial electrical resistance (TEER) measurements must be taken within 6 h of the ending of the exposure, while LDH release and cytokine levels must be measured in conditioned media (either at study termination or when media is changed during the repeated exposure experiments) and apical washings must be collected at the same time as the conditioned medium. The amount of time between the end of test material exposure and the TEER measurements should be as consistent as experimentally feasible and included in the report. H&E, and IHC for p63, MUC5AC, and FOXJ1, should be performed, and morphology changes by light microscopy included in the assessment. Inflammation (cytokine release) should be measured in conditioned medium.	Other assays of cell cytotoxicity may be required if the test item interferes with LDH assessment. The use of multiple biomarkers to assess toxicity is advantageous, as not all chemicals will induce toxicity in the same manner, and therefore improves the chances of picking up a response. It is important that all measures are widely available in many labs and also offer ease of use to aid reproducibility and future conversion into a guideline protocol.
Acute Exposure Controls	Two negative controls tested in parallel—mock air control or vehicle control, as applicable. and incubator control	Other controls may be required: positive control for direct toxicity (e.g., formaldehyde, Triton X-100 or sodium dodecyl sulphate), or lysed cultures for baseline total LDH release. May also be useful to include negative assay controls including substances known to be harmless e.g., saline for direct application or lactose for inhaled applications. The impact of vehicle controls on the condition of the test system may need to be assessed in advance if not known, to minimize impact on study data.
Repeat Dosing	6 h/day for 14 days 3 test concentrations Controls and end-point measurements similar to acute exposure experiments. Note: TEER, LDH release, cytokine levels, and light microscopy observations are non-destructive and must be performed on all wells	The case can be made, as per OECD rodent inhalation test guidelines 413 (90 day) and 412 (28 day), to model a standard working week by dosing in a 5 days on, 2 days rest pattern. 6 h/day is as per in vivo guidelines, but 4 h/day may be sufficient for an in vitro model to be predictive. The inclusion of a repeated exposure scenario is beneficial as it will allow chronic effects to be identified. Repeat exposure can pick up more sensitive changes at dose levels seen as non-toxic in acute tests. For repeat application, we propose to use non-destructive measures such as TEER and LDH release over the time course rather than only at the experiment end. These could also be used to stop the time course early if the barrier had completely broken down or crossed a pre-defined threshold, preventing additional work and maximizing the chance of there being cells present for the H&E and IHC assessments.

## Data Availability

The data presented in this manuscript are available on request from the corresponding author.
